# Prediction of fetal RR intervals from maternal factors using machine learning models

**DOI:** 10.1038/s41598-023-46920-4

**Published:** 2023-11-13

**Authors:** Namareq Widatalla, Mohanad Alkhodari, Kunihiro Koide, Chihiro Yoshida, Yoshiyuki Kasahara, Masatoshi Saito, Yoshitaka Kimura, Ahsan Habib Khandoker

**Affiliations:** 1 Khalifa University, Abu Dhabi, UAE; 2 Next Generation Biological Information Technology, Tohoku University Graduate School of Biomedical Engineering, Sendai, Japan; 3 Department of Biomedical Engineering, Healthcare Engineering Innovation Center, Khalifa University, Abu Dhabi, UAE; 4 Radcliffe Department of Medicine, Cardiovascular Clinical Research Facility, University of Oxford, Oxford, UK; 5 Department of Maternal and Fetal Therapeutics, Tohoku University Graduate School of Medicine, Sendai, Japan; 6 Advanced Interdisciplinary Biomedical Engineering, Tohoku University Graduate School of Medicine, Sendai, Japan; 7 Department of Maternal and Child Health Care Medical Science, Tohoku University Graduate School of Medicine, Sendai, Japan; 8 Department of Obstetrics and Gynecology, Tohoku University Graduate School of Medicine, Sendai, Japan

**Keywords:** Paediatric research, Biomedical engineering

## Abstract

Previous literature has highlighted the importance of maternal behavior during the prenatal period for the upbringing of healthy adults. During pregnancy, fetal health assessments are mainly carried out non-invasively by monitoring fetal growth and heart rate (HR) or RR interval (RRI). Despite this, research entailing prediction of fHRs from mHRs is scarce mainly due to the difficulty in non-invasive measurements of fetal electrocardiogram (fECG). Also, so far, it is unknown how mHRs are associated with fHR over the short term. In this study, we used two machine learning models, support vector regression (SVR) and random forest (RF), for predicting average fetal RRI (fRRI). The predicted fRRI values were compared with actual fRRI values calculated from non-invasive fECG. fRRI was predicted from 13 maternal features that consisted of age, weight, and non-invasive ECG-derived parameters that included HR variability (HRV) and R wave amplitude variability. 156 records were used for training the models and the results showed that the SVR model outperformed the RF model with a root mean square error (RMSE) of 29 ms and an average error percentage (< 5%). Correlation analysis between predicted and actual fRRI values showed that the Spearman coefficient for the SVR and RF models were 0.31 (*P* < 0.001) and 0.19 (*P* < 0.05), respectively. The SVR model was further used to predict fRRI of 14 subjects who were not included in the training. The latter prediction results showed that individual error percentages were (≤ 5%) except in 3 subjects. The results of this study show that maternal factors can be potentially used for the assessment of fetal well-being based on fetal HR or RRI.

## Introduction

The maternal uterine environment functions as an essential substrate for the development of healthy offspring^1^. Previously, it has been well-documented that the overall well-being of a fetus is dependent on the mother^2–4^. Currently, fetal health and development assessments are largely carried out by non-invasive measurements of fetal heart rate (fHR) by using Doppler ultrasound^5,6^. fHR or fetal RR interval (fRRI) is one of the important indicators of fetal well-being and development and it was found to be affected by maternal physiological and psychological conditions^7–9^. For example, it was demonstrated that maternal stress^10^ and exercise^11^ affect fHRs. Also, fHR was found to share a positive correlation with maternal heart rate (mHR) over the long term (24 h) which suggests an interaction between both^12^.

The interaction between maternal and fetal HR is further supported by DiPietro et al.^13^ who analyzed maternal and fetal HRs simultaneously at night and reported that average fHR decreased at night along with mHR during maternal sleep^13^. Another study by Leeuwen et al.^14^ found epochs of synchronization between maternal and fetal HRs when the mother was asked to breathe rhythmically. In our earlier study, we found similarities between maternal and fetal RRI tachograms and we evaluated the same similarities using cross-correlation analysis. We found that the degree of similarities between maternal and fetal RRI tachograms increased with fetal development. All the previously mentioned studies suggest that maternal and fetal HRs are connected.

Previous research that entailed maternal–fetal HR coupling highlighted the potential of studying coupling in the assessment of fetal development and well-being^15–17^. For example, Khandoker et al.^15^ found that coupling patterns of abnormal fetuses were different from control subjects. Also, a study by Wahbah et al.^17^ used coupling to develop a model for the prediction of gestational age (GA). The presence of an association between mHR and fetal development suggests that changes exhibited in mHR can be used for fetal health assessments. However, such assessment is challenging and requires prior knowledge of how and which maternal HR-based factors are related to fetal development.

Identification of possible maternal factors associated with fetal development can be made possible by using artificial intelligence (AI) based techniques. Recently, many AI techniques have proven useful in medical applications and the scope of their application is considered limitless^18^, for example, they have been used for the identification of important features to build predictive models for heart failure^19^ and systolic heart failure survival rate^20^.

Based on the previously mentioned literature, we hypothesize that fRRI can be predicted from maternal features such as maternal HRs, maternal age, and weight. Hence, in this study, we used two supervised learning techniques, support vector regression (SVR) and Random Forest (RF) to investigate the possibility of predicting fRRI from maternal features. We believe that developing such models may assist physicians in assessing fetal development from fHRS.

## Methods

### Data collection and selection

The data described in this study were analyzed retrospectively. 406 pregnant women, who visited Tohoku University Hospital, Japan, were recruited during 2009–2019 after getting their informed consent. The subjects were recruited for different projects that took place at Tohoku University. The same projects involved developing, testing, and validating the fetal abdominal wall-guided fECG Iris Monitor (Atom Medical Corporation, Tokyo, Japan)^21–23^. The study in this manuscript was approved by the Tohoku University Institutional Review Board (Approval Number: 2021-1-133). All methods were performed in accordance with relevant guidelines and regulations (references in Japanese)^24,25^.

Demographics of the mothers were collected such as GA, age, weight, health condition, and medication. Fetal weight and health conditions were recorded as well. During maternal and fetal ECG recording, participants were asked to remain in a supine position and 12 electrodes were attached to their abdominal surface. The recordings lasted for 20 min (sampling frequency: 1 kHz) and one recording (simultaneous maternal and fetal ECG) was collected per subject. The maternal and fetal ECG were recorded simultaneously by using the fetal abdominal wall-guided fECG Iris Monitor (Atom Medical Corporation, Tokyo, Japan, more details about electrode placement and the monitor are found in^23^.

Subjects with missing information regarding maternal weight, GA, or fetal or maternal health were not considered in the analysis. Also, subjects with abnormal fetuses were excluded. Hence, the total number of maternal and fetal ECG records that were considered for analysis in this study was 190, Supplementary Fig. 1 illustrates a summary of data exclusion. Extraction of fECG from maternal ECG (mECG) was conducted by using a MATLAB 2008b code. The code extracts fECG based on blind source separation with reference (BSSR) which is described in detail in^22^. We chose the least optimal window size to perform frequency-based HRV analysis which was 5 min. Hence, extractions of fECG were conducted per 5 min.

We aimed at obtaining two segments of 5 min to calculate the features and average them. From the 190 subjects, extractions of two 5 min segments were possible in 156 subjects (age: 22–44 years old (34 ± 5.3), GA: 19–40 weeks (30 ± 6.2)). Among the 156 pregnant women, 41 had no records of health complications but the rest of the subjects had at least one complication, more details about this can be found in Supplementary Table 1. Extraction of one segment of the same was possible in an additional 14 subjects and this data was used to test the accuracy of our model. We used 5 min segments to accommodate for the very low frequency (VLF) band that requires at least 5 min for proper assessment^26^.

### Model development

In this study, we identified features that we speculated to be useful for fRRI prediction. Oxygen and nutrition delivery to the fetus is dependent on maternal respiration and blood circulation, therefore, we opted for choosing variables that are related to them which included the following maternal features: RRI, HR variability (HRV), R wave amplitude variability (RWAV), age, and weight. Maternal RRI (mRR) was included in the model based on the studies that addressed the presence of correlations between maternal and fetal HRs, the same studies are mentioned in the introduction. Maternal HRV (mHRV), which is an indicator of autonomic nervous system (ANS)^26,27^ activity, was included in the model because it was found to change due to respiration^26^. Further, maternal RWAV (mRWAV) was associated with respiration and stroke volume^28,29^, hence, we included it in the model.

We speculated that the dynamics exhibited in maternal RRI or RW can potentially be used for the prediction of fRRI. We used time and frequency-based HRV parameters for the prediction. With respect to RWAV, we used frequency-based parameters only because we were more interested in RWAV which are known to be affected by respiration^28,29^. Because it is unknown which and how maternal HR or RW-based factors affect fRRI we used the whole frequency power spectrum, and we divided them into the four known bands (VLF, LF, HF and VHF) to get more perspectives on which bands are more predictive of fRRI. Understanding the exact bands that contribute to fRRI prediction can give more insight into the connection between maternal ANS and respiration with fRRI. Maternal age^30^ and weight^31^ were included in the model because they were found to be associated with HRV in adults in previous literature where lower weight and age were associated with higher HRV^30,31^.

### Maternal heart rate variability (HRV) and R wave amplitude variability (RWAV) analysis

To calculate maternal and fetal RRI, maternal and fetal R peaks were detected from non-invasive maternal and fetal ECG, respectively. First, maternal and fetal ECG records were filtered with a 5 Hz high pass filter to remove the baseline noise. After that, the same ECG signals were normalized by dividing the ECG signals with the maximum amplitude value (the maximum value was the highest R peak amplitude value). After that, R peaks were detected in MATLAB 2022a by using the “findpeaks” function. The function provides the amplitude values (RWA values), also, it provides the locations in which the same RWs occurred. Hence, to obtain RRI signals, the values of R wave location were subtracted from each other.

mRRI signals were further used for HRV and RWAV analysis. For mHRV analysis, time and frequency-based analysis was performed. Time-based HRV analysis entailed calculations of average RRI, standard deviation (SD) of NN (normal RR interval) (SDNN) and SD of HR (SDHR). Frequency-based HRV calculations were performed by using the Lomb-Scargle periodogram with the following bands:

VLF^27^: (0.0033–0.04) Hz, LF^27^: (0.04–0.15) Hz, high frequency (HF)^27^: (0.15–0.4) Hz, very high frequency (VHF)^32^: (0.4–0.9) Hz.

Compared to HRV analysis, there is less literature concerning RWAV. In our study, we used the detected RAW values (described above) for RWAV analysis. RWAV analysis was performed by using the same frequency bands mentioned above, VLF, LF, HF and VHF. To distinguish HRV features from that of RWAV, the term RW will be used to refer to RWAV features (RWVLF, RWLF, RWHF, RWVHF). It is worth emphasizing that here, only frequency-based RWAV was calculated as we were interested in capturing ECG respiratory variations.

### Machine learning models and Shapley analysis

MATLAB 2022a was used for developing models and Shapley analysis. Two regression machine learning techniques were used for the prediction of average fRRI which are SVR and RF. SVR, which is an expansion from SV machine (SVM), is a non-parametric supervised learning algorithm that relies on kernel functions to optimize models by finding the best-fit lines with hyperplanes^33^. In MATLAB, SVR utilizes the linear epsilon insensitive (ɛ) SVM regression to find a function *f*(*x*) that does not deviate from response values by more than ɛ^34^. RF is a supervised learning algorithm. RF is an ensemble technique that optimizes models based on many aggregated bootstrap decision trees^35^. For cross-validation, we used leave-one-out in which one data is used for testing and the rest is used for training.

**Figure 1:**
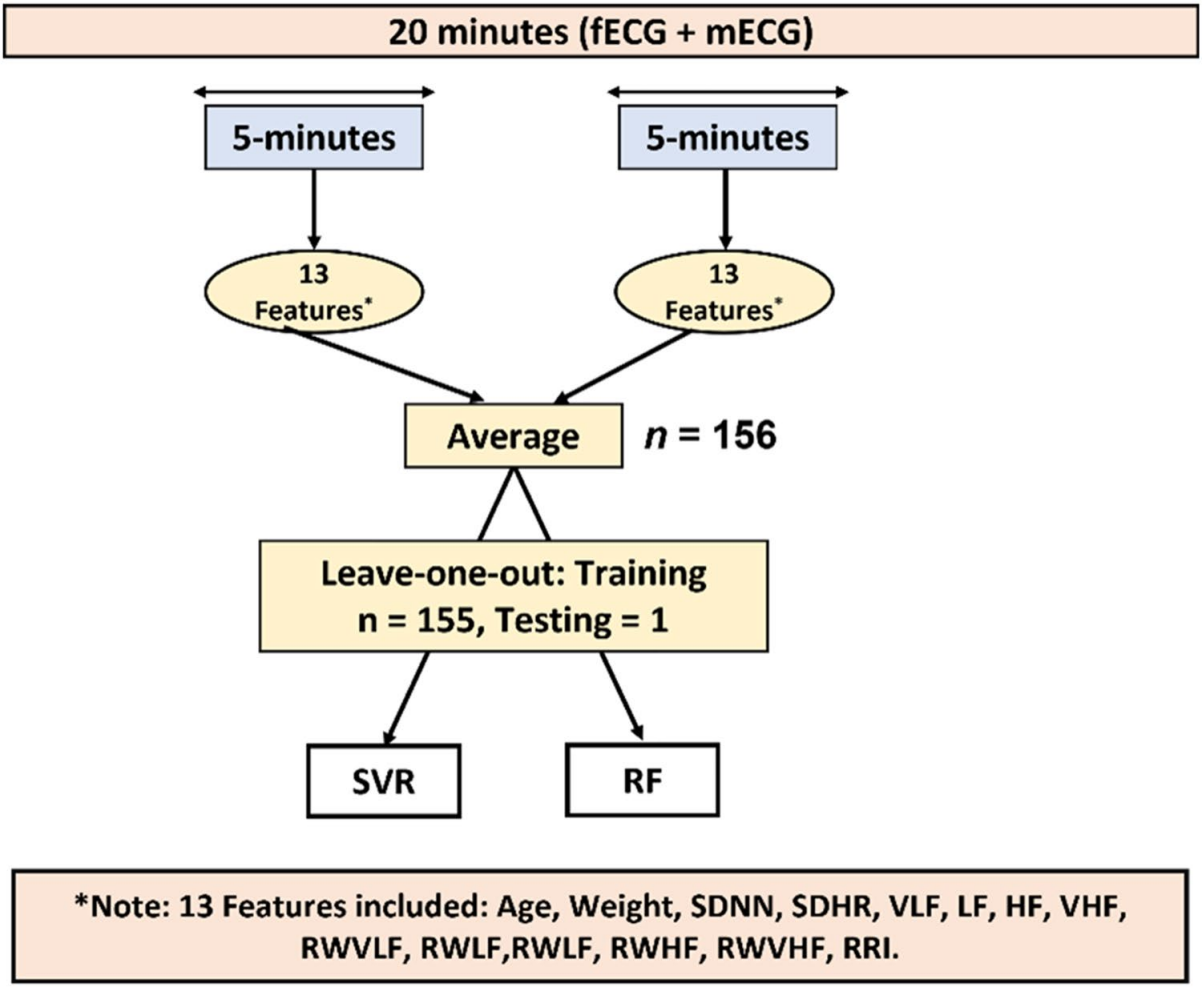
Summary of data analysis. Following extraction of maternal and fetal ECG, 13 features were calculated per 5 min segments. The average from the two 5 min segments was then fed to two models, SVR and RF.

Since SVR and RF are supervised learning algorithms, features should be fed to models for training and testing. In our study, features that were fed to the models were calculated out of the extracted 5 min segments. As was described before, two segments of 5 min were obtained from 156 subjects and the average of features per subject was fed to the models. The total number of maternal features that were fed to the models was 13 which included: 1. Age, 2. Weight, 3. SDNN, 4. SDHR, 5. VLF, 6. LF, 7. HF, 8. VHF, 9. RWVLF, 10. RWLF, 11. RWHF, 12. RWVHF, 13. RRI. Figure 1 shows a summary of the steps that were followed for data analysis.

To get an interpretation of the model, we applied Shapley analysis. Shapleay analysis stems from game theory and basically, it calculates the contribution of a feature to predicting values^36,37^. In other words, Shapley values can provide a general idea about the importance of a feature. Shapley values can be negative or positive and here we considered positive values only because we were interested in the absolute value regardless of the sign. Shapley values were calculated by taking the absolute average from the 156 subjects.

### Model evaluation

To evaluate model performance, root mean square error (RMSE), Bland–Altman (BA)^38,39^ and Spearman^40^ correlation analyses were performed to compare the predicted and model-based values. Correlations with a *p-value* less than 0.05 were considered significant^41^. For further model evaluation, the developed model was tested on 14 subjects that were not used in training the model. Then the predicted fRRI values were compared with the actual values by calculating error percentages per subject by using Eq. (1). Since it is unknown what percentage of error is acceptable for fRRI prediction, we defined an error value equal to or less than 5% to be acceptable as was identified in previous studies^42,43^.

1 $$Error \left( \% \right) = \frac{{\left| {Predicted \,fRRI - Actual\, fRRI} \right|}}{Actuall \,fRRI} \times 100$$

## Results

### Summary of the features

Table 1 shows the mean and SD values for the maternal features and fRRI. The first 13 maternal features in the table were fed to the SVM and RF models to predict the 14^th^ feature, fRRI.

**Table 1:** Mean and SD values of maternal features and fRRI.

Feature	(Mean ± SD)
RRI (ms)	763 ± 114
SDNN (ms)	34 ± 14
SDHR (bpm)	3.6 ± 1.3
VHF (ms^2^/Hz)	44 ± 146
HF (ms^2^/Hz)	254 ± 356
LF (ms^2^/Hz)	229 ± 223
VLF (ms^2^/Hz)	664 ± 565
RWVHF (ms^2^/Hz)	0.016 ± 0.012
RWHF (ms^2^/Hz)	0.056 ± 0.040
RWLF (ms^2^/Hz)	0.019 ± 0.011
RWVLF (ms^2^/Hz)	0.037 ± 0.025
Age (years)	34 ± 5.3
Weight (Kg)	60 ± 8.4
fRRI (ms)	412 ± 24

*SD* standard deviation, *RRI* RR interval, *SDNN* SD of normal to normal beat, *SDHR* SD of heart rate, *bpm* beats per minute, *VHF* very high frequency, *HF* high frequency, *LF* Low frequency, *VLF* very low frequency, *RWVHF* R wave very high frequency, *RWHF* R wave high frequency, *RWLF* R wave low frequency, *RWVLF* R wave very low frequency, *fRRI* fetal RRI.

### Comparison between SVR and RF performance

**Figure 2:**
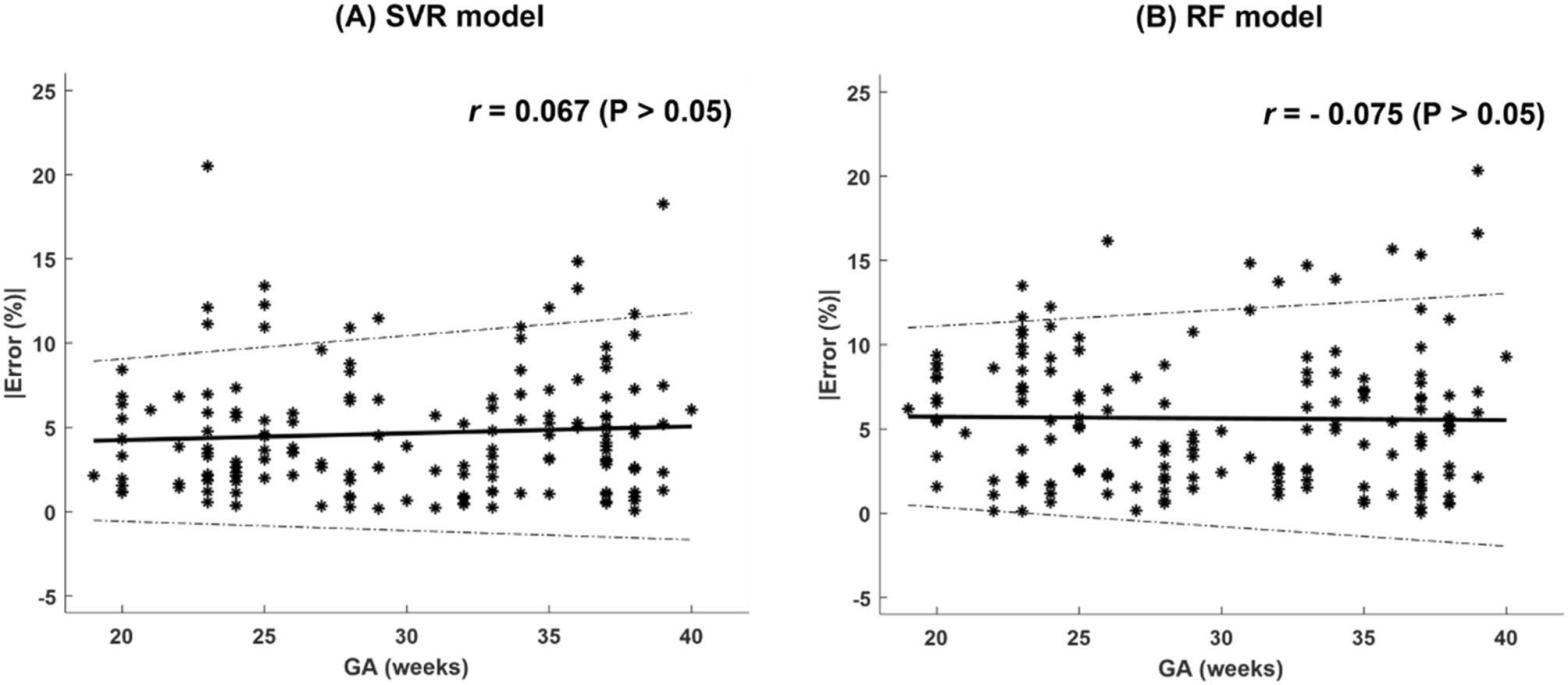
Absolute error percentage versus gestational age (GA). (**A**) support vector regression (SVR) model. (**B**) Random forest (RF) model.

We investigated the change in accuracy of the prediction with GA by calculating the Spearman correlations between the Error percentages and GA, Fig. 2. In Fig. 2, the accuracy of the model did not change with GA as the correlation coefficients were almost zero per model: SVR (*r* = 0.067 (*p* > 0.05), Fig. 2A) and RF (*r* = − 0.057 (*p* > 0.05), Fig. 2B).

**Figure 3:**
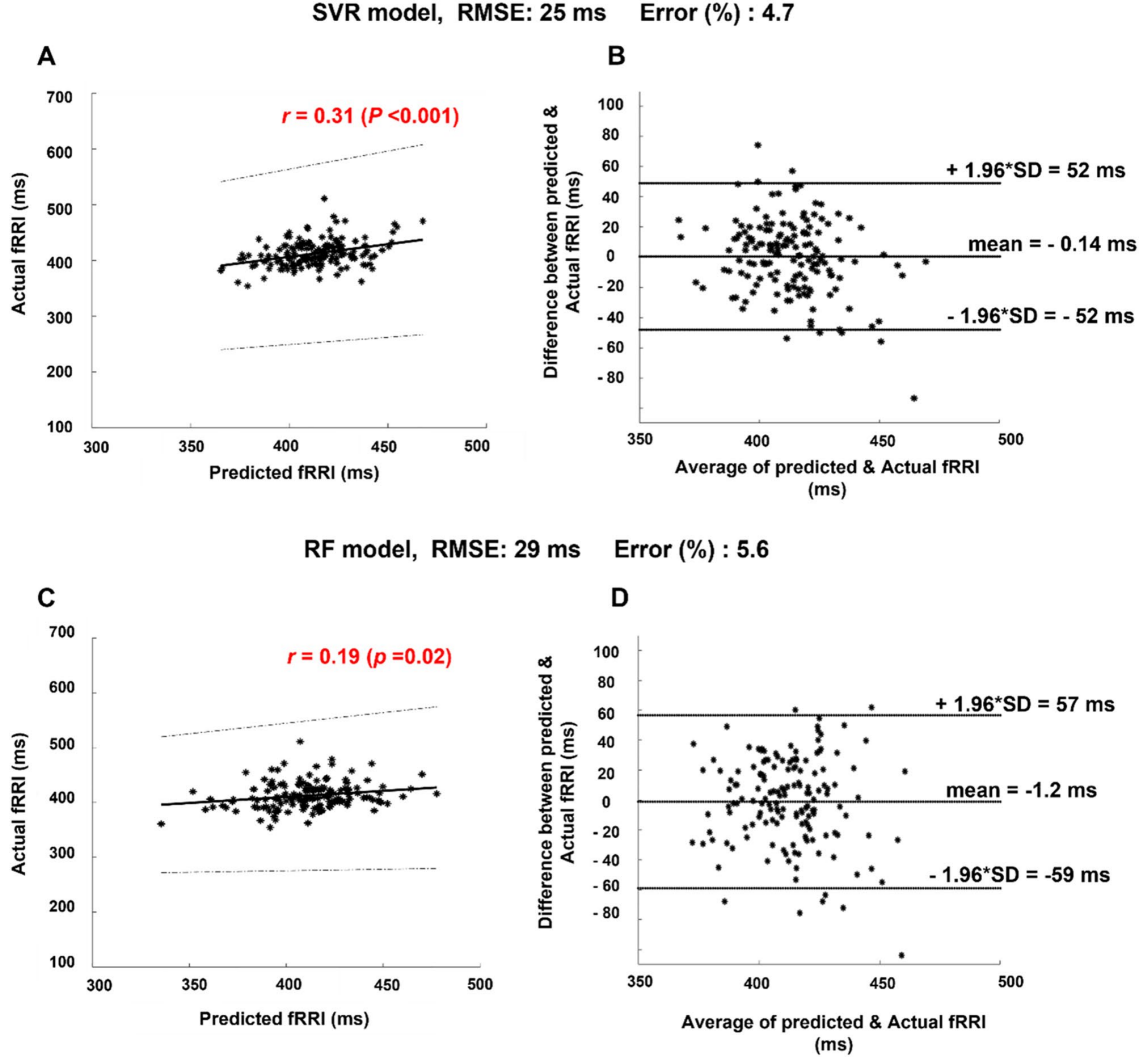
Bland Altman (BA) and correlation analysis. (**A**) and (**B**) show the results of the support vector machine (SVR) model and (**B**) and (**C**) show the same for the random forest (RF) model. The limits of agreement of BA plot, root mean square error (RMSE) and error percentage are lower in the SVR model. Also, the Spearman correlation coefficient (*r*) value of the SVR model is higher than that of the RF model. The latter facts show that the SVR model was more effective in the prediction of fRRI values.

The results of model validation revealed that the SVR model is more accurate compared to the RF model, Fig. 3. The latter is because of the lower REMSE value for the SVR model (RMSE = 25 ms) compared to the RF model (RMSE = 29 ms). Furthermore, the results of Spearman correlation analysis showed a weak but significant correlation for the RF model (*r* = 0.19 (*P* < *0.05*), Fig. 3A) and a moderate correlation for the SVR model (*r* = 0.31, *p* < *0.05*, Fig. 3C). BA plots (Fig. 3B,D) show that the percentage of points that were within the limits of agreement was around 95%, nevertheless, the upper (52 ms) and lower (− 52 ms) boundaries in (Fig. 3B) were narrower than (Fig. 3D, upper boundary: 57 ms, lower boundary: − 59 ms) implying higher accuracy for the SVR model. In BA plots, it is revealed that both models generally underestimated the predicted values and according to the mean values (SVR:− 0.14 ms, RF: − 1.2 ms), it is revealed that SVR has higher accuracy due to the lower mean value compared to the RF model. Due to the higher accuracy of the SVR model, it was used further for Shapley analysis and prediction of fRRI values that were not involved in the training.

### Shapley analysis

**Figure 4:**
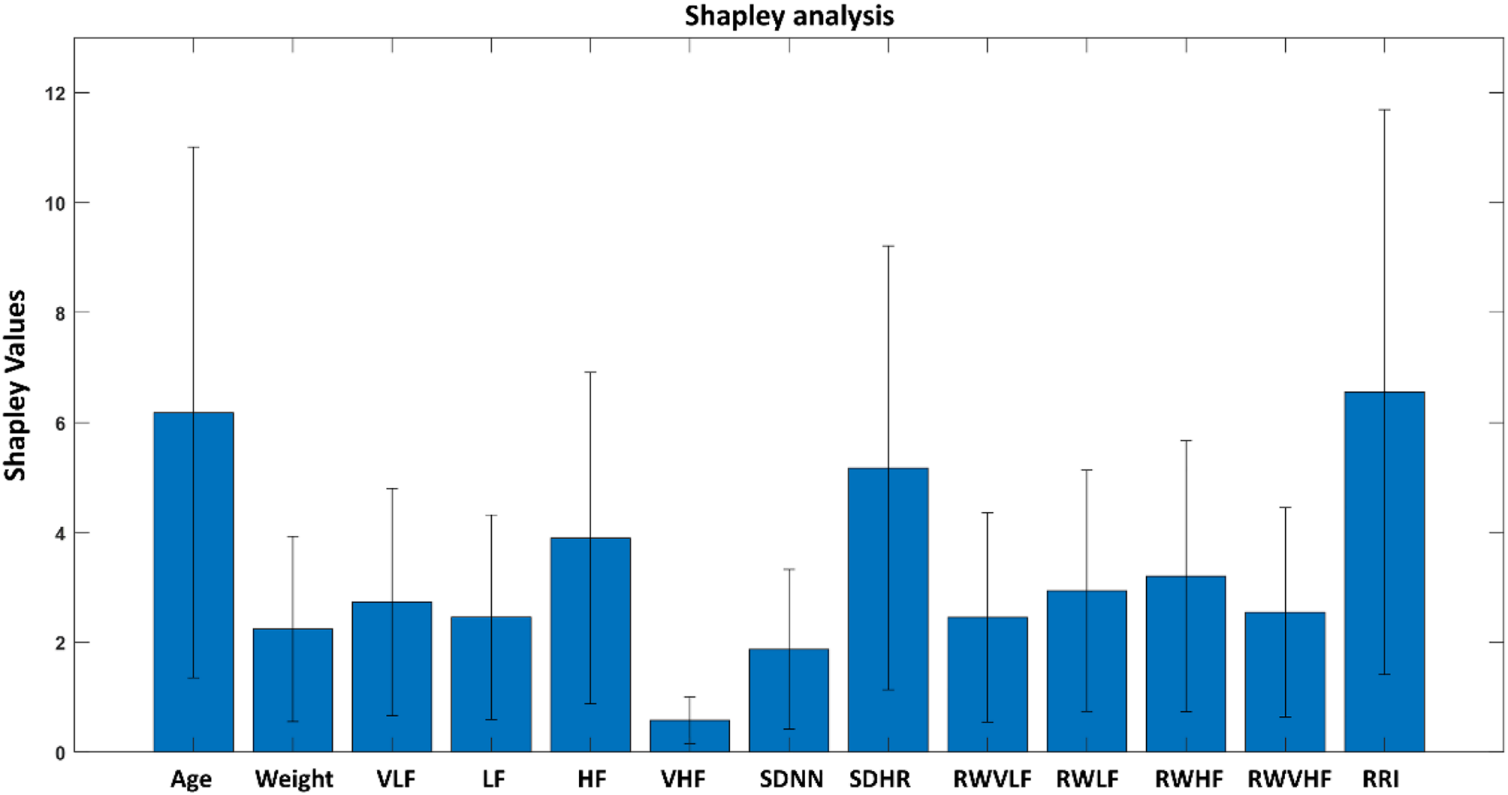
Average Shapley values with 95% confidence interval (CI): Shapley analysis shows that age, HF, SDHR and RRI had the highest impact on average fetal RR interval (fRRI) prediction whereas very high frequency (VHF) had the lowest impact.

The absolute average values of Shapley along with the 95% confidence interval are shown in Fig. 4. In the figure, it is revealed that among the 13 features, age, HF, SDHR and RRI had a major effect on fRRI predictions whereas VHF was found to have the lowest impact.

### Model validation on test subjects

After developing the model, we attempted to predict fRRI values from the 14 subjects that were not considered in training the model. The results of prediction per subject are listed in Table 2. Error percentages were calculated to estimate the accuracy of prediction and as the table shows, they were less than or equal to 5% in general except in 3 cases (subject #4, 5 &13).

**Table 2:** SVR model prediction of fRRIs.

Subject number (GA (weeks))	Maternal age (years)	Maternal complication	Actual fRRI (ms)	Predicted fRRI (ms)	Error (%)
1 (28)	43	Uterine/appendix disease	445	434	2.5
2 (32)	43	Uterine/appendix disease	434	420	3.3
3 (37)	30	Uterine/appendix disease	412	416	0.9
**4 (25)**	**39**	Uterine/appendix disease	**408**	**435**	**6.5**
**5 (29)**	**35**	Uterine/appendix disease	**385**	**438**	**14**
6 (37)	31	Central nervous system disease (CNS)	370	388	4.9
7 (23)	29	None	419	418	0.4
8 (26)	30	None	383	370	3.0
9 (29)	29	Autoimmune disease	432	422	2.2
10 (37)	42	CNS, Bone muscle diseases, Multiple sclerosis	446	431	3.1
11 (38)	27	Mental illness	409	429	5.0
12 (38)	32	Mental illness	407	420	3.1
**13 (39)**	**45**	**Respiratory disease, uterine/appendix disease**	**502**	**410**	**18**
14 (33)	32	Psoriasis vulgaris	418	435	4.1

Values with error % higher than 5% are in bold.

## Discussion

In this study, we developed two models to predict fRRI from 13 maternal features and a comparison analysis between SVR and RF models revealed that SVR outperformed RF in predicting fRRI (Fig. 3). Due to the better performance of SVR, we used it further in Shapley analysis (Fig. 4) and prediction of fRRI from the 14 subjects that were not used in the training (Table 2). In Fig. 4, it is demonstrated that between age and weight, age had a stronger effect on fRRI prediction. Further, the figure shows that among the ECG-derived parameters, maternal RRI, SDHR, and HF had the highest impact on fRRI prediction.

As was mentioned in the introduction, the effect of maternal HR or RRI on fHR was documented in previous studies^12,44,45^. However, the association of maternal age, HF and SDHRs with fHR is less documented. In previous studies^4,46^, it was addressed that advanced maternal age was associated with preterm delivery and small for gestational age^46^, but the mechanisms related to this are not fully understood yet. In our study, we show that maternal age has an impact on fRRI (Fig. 4), which could be one of the pathways by which maternal age affects fetal development. At advanced age, there is a tendency for HRV to decrease and for blood pressure to increase which may lead to health complications without proper management^47^. As a result, fRRI is expected to get affected by age-related changes in maternal cardiac functionality. The effect of maternal HRV on fRRI is still to be understood but previously it was demonstrated that maternal breathing affected maternal and fetal HR coupling^9^. Respiration is known to affect HRV, especially the HF band^26,27^, hence, the associations of fRRI with maternal SDHR and HR that were found in our study could be respiratory mediated, but more research is needed to validate this.

Table 2 shows that the model was generally effective in predicting fRRI since the error percentages were less than or equal to 5% except in three cases, subjects #4, 5 & 13. We expected to find deviations between the actual and predicted values because we speculate that maternal effect on fRRI is partial and not dominant because fRRI is controlled by many physiological processes such as fetal behavioral states^48^. Also, we expect that maternal complications may affect the prediction accuracy. It is worth mentioning that, due to the limited knowledge in the field, it is unknown if a particular measured fRRI value indicates a norm or not. Hence, training predictive models to distinguish a norm could assist physicians in the assessment of fetal health. Model-based prediction of fRRI from maternal condition is yet to be explored and so far, it is unknown which machine learning technique could be suitable for this type of application. Therefore, in this study, we opted for using two of the commonly used techniques for regression, SVR and RF.

The correlations between predicted fRRI and actual fRRI (Fig. 3) were significant but the coefficient values were lower than 0.5. we expect that the correlation could be enhanced if the training set was composed of mothers with no abnormalities and similar age and weight. Also, we expect the correlation values could be enhanced if additional maternal features were added such as blood pressure.

Although our study shows that our SVR model showed an acceptable prediction based on significant correlation (Fig. 3) and error percentages (Table 2), it has several limitations. Our retrospective design constitutes a major limitation. As was mentioned in Methods, pregnant women had complications and we expect that such complications affected the accuracy of the model. Also, we expect that inclusion of more maternal-related information may enhance model accuracy such as maternal blood pressure, respiratory rate, and height. Maternal height was not available for all subjects; hence, it was not included as a feature in our model.

In conclusion, we discussed two models, SVR and RF, to predict fRRI from maternal factors. The predicted fRRI values correlated significantly with actual fRRI demonstrating a dependency between the mother and fetal development. The results reported in this study suggest that fetal development and health assessment can be enhanced by integrating maternal conditions.

### Supplementary Information

Supplementary Information.

## Data Availability

Data are available upon a proper request by contacting Dr Yoshiyuki Kasahara: kasa@med.tohoku.ac.jp.
