# Supplementary material for: Prediction of fetal RR intervals from maternal factors using machine learning models
Source: Sci Rep. 2023 Nov 13;13:19765. doi: 10.1038/s41598-023-46920-4 (PMC10643643; doi:10.1038/s41598-023-46920-4)
Supplement: Supplementary file 1 — Supplementary Information. [file 41598_2023_46920_MOESM1_ESM.docx]

**Prediction of fetal RR interval from maternal factors using machine learning models**

Namareq Widatalla, Mohanad Alkhodari, Kunihiro Koide, Chihiro Yoshida, Yoshiyuki Kasahara, Masatoshi Saito, Yoshitaka Kimura, Ahsan Khandoker

**Supplementary Material**

Supplementary Table 1: Detailed information about the participants (n=156)

| **Maternal Condition** | **Number of cases** | **GA (weeks)**  median (min-max) | **Age (years)**  median (min-max) |
| --- | --- | --- | --- |
| Normal | 41 | 32 (20 – 39) | 34 (22 – 44) |
| Central nervous system (CNS) disease | 10 | 34 (23 – 38) | 32 (22 – 40) |
| Essential hypertension | 2 | 23 - 27 | 29 |
| Blood disease | 5 | 28 (23 – 38) | 41 (27 – 41) |
| Thyroid disease | 1 | 28 | 38 |
| Mental Illness | 11 | 31 (22 – 39) | 35 (27 – 40) |
| Respiratory disease | 9 | 31 (23 – 40) | 29 (27 – 38) |
| Gestational Diabetes | 5 | 33 (19 – 38) | 31 (23 – 34) |
| Uterine/appendix disease (UAD) | 14 | 24 (20 – 38) | 35 (29 – 43) |
| Autoimmune disease | 9 | 32 (25 – 39) | 33 (29 – 36) |
| Heart disease | 3 | 25 (23 – 26) | 26 (25 – 33) |
| Placenta previa | 6 | 34 (20 -36) | 37 (31 – 40) |
| Bone and muscle system disease | 2 | 28 & 32 | 33 & 33 |
| Kidney disease | 1 | 28 | 32 |
| Pre-eclampsia | 1 | 37 | 27 |
| Diabetes | 1 | 37 | 32 |
| Osler's disease | 1 | 36 | 32 |
| Cervical weakness | 1 | 39 | 30 |
| Blood disease & gestational diabetes | 1 | 33 | 41 |
| Cervical weakness & gestational diabetes | 1 | 22 | 41 |
| Kidney and respiratory diseases | 1 | 37 | 32 |
| UAD & autoimmune disease | 6 | 31 (26 – 38) | 33 (28 – 34) |
| UAD & Thyroid disease | 2 | 32 & 39 | 26 & 37 |
| UAD & digestive system disease | 1 | 20 | 41 |
| Thyroid and blood diseases | 1 | 23 | 37 |
| Thyroid and autoimmune disease | 1 | 24 | 30 |
| Autoimmune disease and placenta previa | 1 | 37 | 39 |
| Respiratory disease and mental illness | 1 | 20 | 38 |
| Mental illness and placenta previa | 1 | 37 | 32 |
| UAD & Mental illness | 3 | 34 (20 – 38) | 34 (25 – 34) |
| UAD & respiratory disease | 1 | 20 | 38 |
| UAD & Bone and muscle system disease | 1 | 23 | 44 |
| UAD & Urinary system disease | 1 | 38 | 39 |
| Gestational diabetes & placenta previa | 1 | 33 | 30 |
| UAD & gestational diabetes | 1 | 26 | 34 |
| Respiratory disease & placenta previa | 2 | 35 & 37 | 35 & 39 |
| UAD & essential hypertension & thyroid disease | 1 | 38 | 41 |
| UAD & placenta previa | 1 | 33 | 32 |
| UAD & CNS | 1 | 23 | 35 |
| Placenta previa & cervical weakness | 1 | 25 | 35 |
| Heart disease and thyroid disease | 1 | 20 | 43 |
| Heart disease & respiratory disease and CNS | 1 | 2 | 27 |


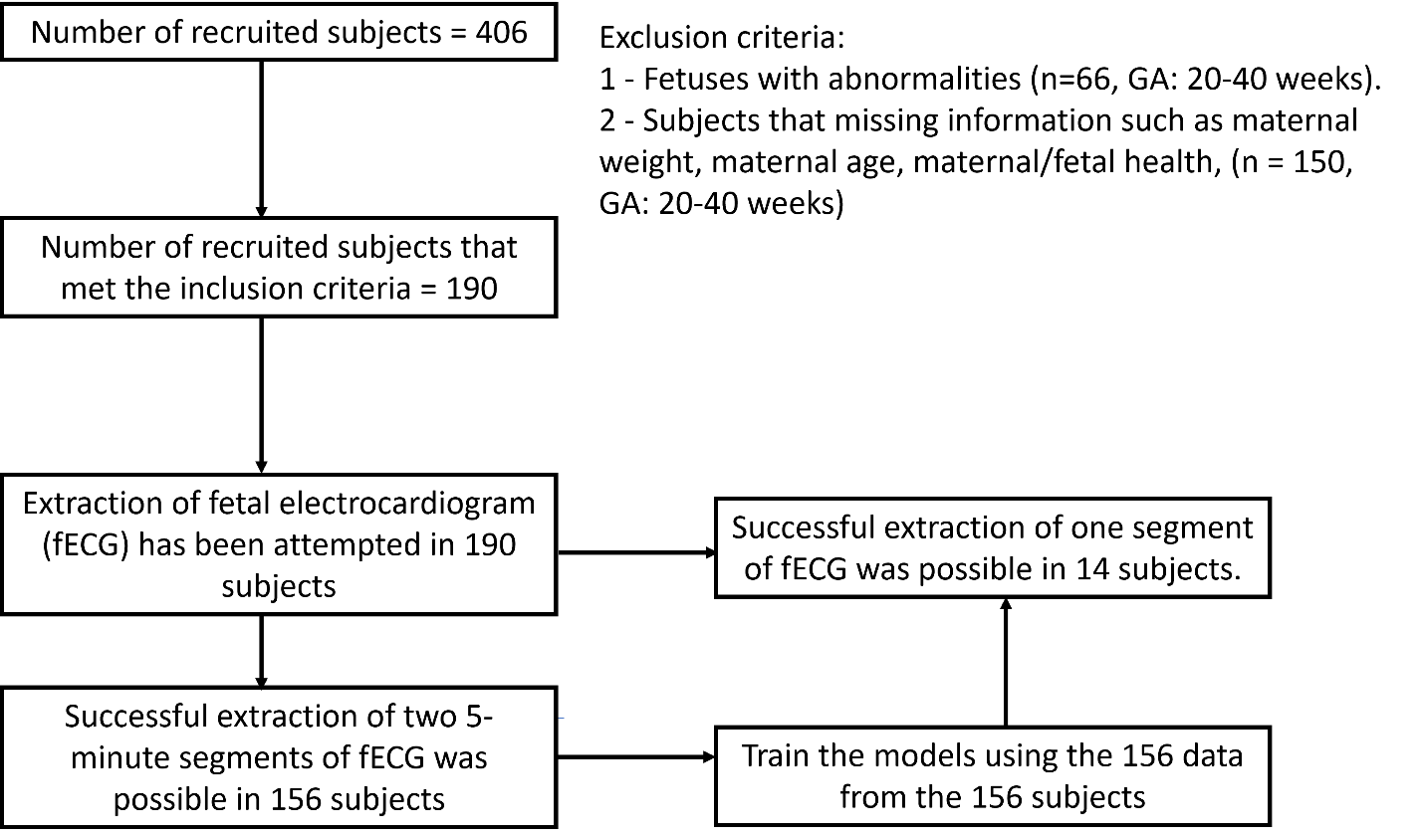


**Supplementary Figure 1: Summary of data inclusion and analysis**
